# Higher Institutional Volume Reduces Mortality in Reoperative Proximal Thoracic Aortic Surgery

**DOI:** 10.1055/s-0040-1713860

**Published:** 2020-11-05

**Authors:** Nicholas J. Shea, Alex M. D'Angelo, Antonio R. Polanco, Philip Allen, Joseph E. Sanchez, Paul Kurlansky, Virendra I. Patel, Hiroo Takayama

**Affiliations:** 1 Department of Surgery, New York-Presbyterian Hospital, Columbia University Aortic Surgery Center, Columbia University Irving Medical Center, New York, New York

**Keywords:** reoperation, aortic surgery, aortic aneurysm, aortic dissection

## Abstract

**Objective** This study aims to determine the impact of institutional volume on mortality in reoperative proximal thoracic aortic surgery patients using national outcomes data.

**Methods** The Nationwide Inpatient Sample was queried from 1998 to 2011 for patients with diagnoses of thoracic aneurysm and/or dissection who underwent open mediastinal repair. A total of 103,860 patients were identified. A total of 1,430 patients had prior cardiac surgery. Patients were further stratified into groups by institutional aortic volume: low (<12 cases/year), medium (12–39 cases/year), and high (40+ cases/year) volume. Multivariable risk-adjusted analysis accounting for emergent status and aortic dissection among other factors was performed to determine the impact of institutional volume on mortality.

**Results** Overall mortality was 12% in the reoperative population. When the redo cohort was divided into tertiles, high-volume group had a 5% operative mortality compared with 9 and 15% for the medium- and low-volume groups, respectively. Multivariable analysis revealed that patients operated on at low- (odds ratio [OR] = 5.0, 95% confidence interval [CI]: 2.6–9.6, *p* < 0.001) and medium-volume centers (OR = 2.1, 95% CI: 1.1–4.2, *p* = 0.03) had higher odds of mortality when compared with patients operated on at high-volume centers.

**Conclusions** High-volume aortic centers can significantly reduce mortality for reoperative aortic surgery, compared with lower volume institutions.

## Introduction

In the current era, first time proximal aortic surgical operations (e.g., root, ascending, and arch procedures) are being performed with low operative mortality, with some institutions reporting mortality less than 5%. 1 While these excellent outcomes in first time patients are encouraging, an increasing number of reoperative aortic procedures are being performed. Patients with prior open aortic and cardiac operations are aging and requiring surgery for aortic disease including progressive aneurysmal degeneration, new dissections, or infectious complications. 2 3 Several institutions have reported their outcomes in this reoperative proximal aortic surgical population, with some referral centers reporting operative mortality as low as 4%, while others report mortality as high as 19%. 4 5 6 7 8 9 10 11 12 13 14 15 Anecdotally, higher volume centers experience lower mortality, although the relationship between surgical volume and mortality in this complex surgical population remains largely unexamined.

In this study, we hypothesize that outcomes in the reoperative proximal aortic surgical population vary with institutional volume. To test this hypothesis, we used national outcomes data to evaluate the impact of institutional annual reoperative proximal thoracic aortic surgical (i.e., root, ascending, or arch procedure) volume on operative mortality among patients in the United States with prior cardiac or aortic surgery via sternotomy.

## Materials and Methods

### Data Source

This study is a retrospective cohort analysis of patients who underwent reoperative proximal thoracic aortic surgery in the United States in the years 1998 to 2011 selected from the Healthcare Cost and Utilization Project National Inpatient Sample (HCUP-NIS), sponsored by the Agency for Healthcare Research and Quality. HCUP-NIS is a 20% stratified probability sample including approximately 8 million acute hospital stays annually from more than 1,000 hospitals in 42 states. It is the largest all-payer inpatient care observational cohort in the United States, representing approximately 90% of all hospital discharges. Each record in the database represents an inpatient stay and includes patient demographics, principal diagnoses, comorbidities and complications, and procedures coded according to the International Classification of Diseases, Ninth Revision, Clinical Modification (ICD-9-CM). Weights based on sampling probabilities for each stratum are used in the analysis to ensure that the hospitals studied are representative of all U.S. hospitals. This study was granted exemption from institutional review board approval at our institution because HCUP-NIS is a public database with no personal identifying information.

### Patient Selection

The NIS database was queried from 1998 to 2011, and all patients with a history of prior heart surgery (ICD-9-CM code V15.1) and a procedure code for thoracic aortic replacement (ICD-9-CM codes 38.34, 38.35, 38.45) were identified. Patients were only included in the final analysis if they had diagnosis codes for thoracic aortic aneurysm (ICD-9-CM codes 441.1 or 441.2) or dissection (ICD-9-CM code 441.01). In an attempt to remove patients undergoing surgery for descending thoracic aortic aneurysms, patients were excluded if they did not have a diagnosis code for cardiopulmonary bypass (ICD-9-CM 39.61), cardioplegia (ICD-9-CM 39.63), hypothermia (ICD-9-CM 39.62), or concomitant valve or coronary bypass procedures. A total of 1,430 patients were identified meeting our inclusion criteria and represent the cohort for this study.

### Volume

A unique hospital identifier is provided by the NIS to identify the institution from which a patient was discharged. Using this unique identifier, all patients included in our cohort had the institution performing their operation identified. The total annual volume of proximal thoracic aortic surgeries performed at each individual institution was determined over the course of the study period using the same inclusion criteria outlined above. After annual volume was determined for the relevant institutions, they were stratified into tertiles by annual volume. High-volume centers (HVCs) were defined as those centers performing 40+ thoracic aortic operations/year (lower limit of upper tertile), medium-volume centers (MVC) were defined as those performing 12 to 39 thoracic aortic operations/year, and low-volume centers (LVC) as those performing <12 thoracic aortic operations/year (upper limit of lower tertile). Further details of redo aortic case volume based on each volume tertile can be found in Table 1 . A comparison of clinical characteristics and demographics is detailed in Tables 2 and 3 .

**Table 1:** Redo aortic case volume details based on institutional volume tertile groupings

Variable	Overall	HVC	MVC	LVC
Mean (SD)	57.8 (38.4)	83.8 (30.4)	26.6 (7.7)	8.2 (3.1)
Range	0.36–155.9	45.4–155.9	12–39.7	0.36–11.8
Median [IQR]	50.6 [26.8, 77.3]	75.3 [64.5, 92.1]	26.8 [20.2, 33.8]	9.4 [5.5, 11]
Total number of centers	98	21	39	38

Abbreviations: HVC, high-volume center; IQR, interquartile range; LVC, low-volume center; MVC, medium-volume center; SD, standard deviation.

**Table 2:** Patient characteristics comparison between volume tertile groupings

Variable	HVC ( *n* = 534) % (no.)	MVC ( *n* = 503) % (no.)	LVC ( *n* = 392) % (no.)	*p* -Value
Age (mean) in y	55.6	56.9	46.1	< **0.001**
Female gender	17.6 (94)	27.4 (138)	29.8 (117)	< **0.001**
Chronic kidney disease	1.9 (10)	6.2 (31)	1 (4)	< **0.001**
Chronic obstructive pulmonary disease	14.6 (78)	8.9 (45)	6.4 (25)	< **0.001**
Diabetes	9.3 (50)	15.5 (78)	14.3 (56)	**0.008**
Cerebrovascular disease	1.7 (9)	4.8 (24)	1.3 (5)	**0.001**
Peripheral vascular disease	0.9 (5)	7.5 (38)	1.3 (5)	< **0.001**
Hypertension	60.7 (325)	49 (247)	49.9 (196)	< **0.001**
Operation performed at teaching institution	89.2 (477)	86.3 (435)	76.0 (294)	< **0.001**

Abbreviations: HVC, high-volume center; LVC, low-volume center; MVC, medium-volume center.

**Table 3:** Operative characteristic comparison between volume tertile groupings

Variable	HVC ( *n* = 534) % (no.)	MVC ( *n* = 503) % (no.)	LVC ( *n* = 392) % (no.)	*p* -Value
Concomitant coronary surgery	26.0 (139)	17.9 (90)	17.8 (70)	**0.001**
Concomitant valve surgery	42.5 (227)	51.2 (258)	36.0 (141)	< **0.001**
Elective presentation	60.9 (301)	68.1 (307)	62.1 (220)	0.216
Urgent presentation	17.6 (87)	14.4 (65)	16.7 (59)	–
Emergent presentation	21.5 (106)	17.5 (79)	21.2 (75)	–
Aortic dissection	26.8 (143)	26.6 (134)	20.7 (81)	0.064
Thoracic aneurysm	75.1 (534)	73.4 (370)	79.3 (311)	0.113

Abbreviations: HVC, high-volume center; LVC, low-volume center; MVC, medium-volume center.

### Study Endpoints and Statistical Analysis

**Fig. 1:**
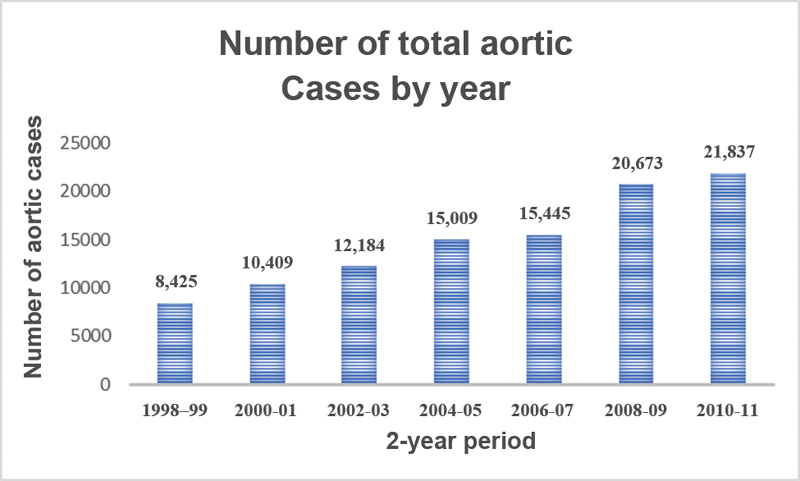
Increasing total redo aortic volume over from 1998 to 2011.

The primary endpoint of this study was in-hospital mortality. A comparison of in-hospital clinical outcomes is detailed in Table 4 . Univariate analysis was performed using Pearson's Chi-square test for categorical variables and analysis of variance for continuous variables. Multivariable analysis was performed using a binary logistic regression model to determine the impact of annual aortic volume on in-hospital mortality. Stepwise construction was not performed so as not to overfit the data. All baseline characteristics were included in the multivariable model. Annual aortic volume was placed into the model as a categorical variable corresponding to the tertile of the institution at which a patient had surgery. HVCs were used as a reference group. In-hospital mortality was chosen as the dependent variable in this model. Results of the multivariable analyses are represented as odds ratios (OR) with 95% confidence intervals (CIs). A multivariable subanalysis was performed on patients who presented for elective surgery using a similar analysis. Both multivariable analyses are detailed in Table 5 . The number of redo proximal thoracic aortic operations over the study period by year is detailed in Fig. 1 . All analyses were performed with SPSS for Macintosh, Version 24 (IBM Corporation, Armonk, NY). All tests were two-sided, with statistical significance set at a value of *p* < 0.05.

**Table 4:** Comparison of in hospital morbidity and mortality between volume tertile groupings

Variable	HVC ( *n* = 534) % (no.)	MVC ( *n* = 503) % (no.)	LVC ( *n* = 392) % (no.)	*p* -Value
Mortality	4.7 (25)	9.1 (46)	14.8 (58)	< **0.001**
Cardiac complications	12.5 (67)	15.1 (76)	10.2 (40)	0.090
Acute kidney injury	13.6 (73)	11.3 (57)	16.8 (66)	0.062
Stroke	6.4 (34)	5.8 (29)	1.3 (5)	**0.001**
Gastrointestinal complications	7.1 (38)	1.0 (5)	4.8 (19)	< **0.001**
Pulmonary embolism	0.9 (5)	1.8 (9)	0.0 (0)	**0.026**
Urinary complications	11.2 (60)	10.5 (53)	8.9 (35)	0.516
Respiratory failure	1.7 (9)	1.8 (9)	0.0 (0)	**0.032**

Abbreviations: HVC, high-volume center; LVC, low-volume center; MVC, medium-volume center.

**Table 5:** Multivariable analysis of impact of volume on mortality

Variable	Entire cohort		Elective cohort	
Volume tertile	Odds ratio (95% confidence interval [CI])	*p* -Value	Odds ratio (95% CI)	*p* -Value
Low volume	5.0 (2.62–9.59)	<0.001	5.7 (1.21–26.7)	**0.03**
Medium volume	2.1 (1.10–4.19)	0.03	1.9 (0.48–7.36)	0.4
High volume	1	1	1	1

## Results

### Patient and Operative Characteristics

Patients operated on at LVC were significantly younger than patients operated on at HVC (mean age, 46.1 vs 55.6 years). Further, LVC patients had significantly lower frequency of chronic lung disease (6.4 vs. 14.6%), were less likely to be operated on at teaching institutions (79 vs. 86%), and were more likely to be female (29.8 vs. 17.6%). A full comparison of patient characteristics can be found in Table 2 .

Patients operated on at HVCs were more likely to undergo concomitant coronary surgery (26 vs. 18%) and valve surgery (42.6 vs. 36%) when compared with patients operated on at LVC. There was not a significant difference in the acuity of presentations between the tertile groupings. Lastly, there was a trend toward a significant difference in frequency of proximal aortic dissection between the HVC and LVC groups, 26.8 versus 20.7%, respectively. A full comparison of operative characteristics can be found in Table 3 .

### In-Hospital Outcomes

In the unadjusted comparison of in-hospital outcomes between tertile groupings, HVC patients had an in-hospital mortality of 4.7%, while the in-hospital mortality for MVC and LVC were 9 and 15%, respectively. HVC had higher stroke and respiratory failure rates when compared with LVC. Rates of acute kidney injury, cardiac complications, and urinary complications were not significantly different among the groups. Gastrointestinal complications were highest among the HVC group. Pulmonary embolism was highest among the MVC group. A full comparison of in-hospital outcomes can be found in Table 4 .

### Impact of Volume

In the multivariable analysis of the entire cohort, annual aortic volume tertile was a significant predictor of in-hospital mortality. HVCs were used as a reference in the analysis. When compared with HVC, patients had five times the odds of in-hospital mortality (OR = 5.01, 95% CI: 2.62–9.59, *p* < 0.001) if their operations were performed at LVC, and patients had two times the odds of in-hospital mortality if their operations were performed at an MVC. Other significant predictors of in-hospital mortality in this cohort were cerebrovascular disease, peripheral vascular disease, and emergent and urgent presentations, as detailed in Table 6 .

**Table 6:** Other significant predictors of mortality

Variable	Odds ratio	95% confidence interval	*p* -Value
Cerebrovascular disease	4.4	1.2–16.3	**0.03**
Peripheral vascular disease	3.4	1.3–9.0	**0.02**
Emergent presentation	3.8	2.1–7.2	< **0.001**
Urgent presentation	3.0	1.5–6.0	**0.003**
Elective presentation	1	1	1

Given the challenges in transferring urgent and emergent patients to higher volume centers, an additional analysis of only patients presenting electively was performed. About 915 of the original 1,430 patients were included in the elective only analysis. In this subanalysis, when compared with patients operated on at HVC, patients operated on at LVC also had five times the odds of in-hospital mortality (OR = 5.69, 95% CI: 1.21–26.7, *p* = 0.03). There was no significant difference in the odds of in-hospital mortality between HVC and MVC (OR = 1.8, 95% CI: 0.48–7.36, *p* = 0.36).

### Trends in Aortic Surgery

**Fig. 2:**
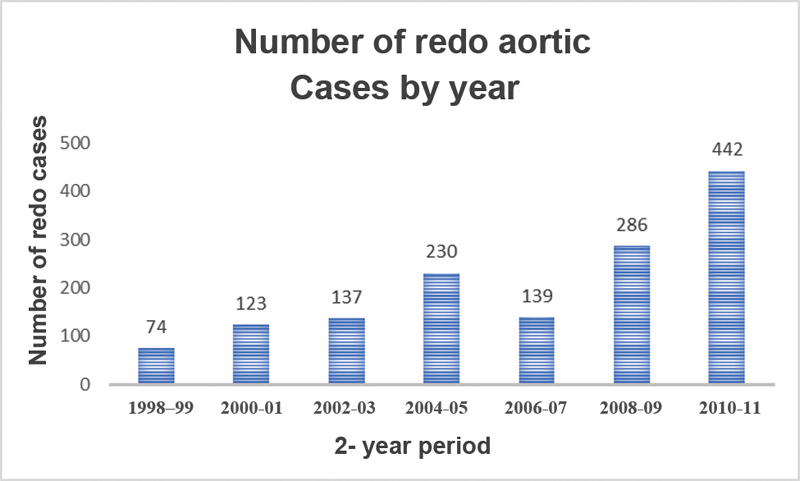
Increasing total aortic volume from 1998 to 2011.

**Fig. 3:**
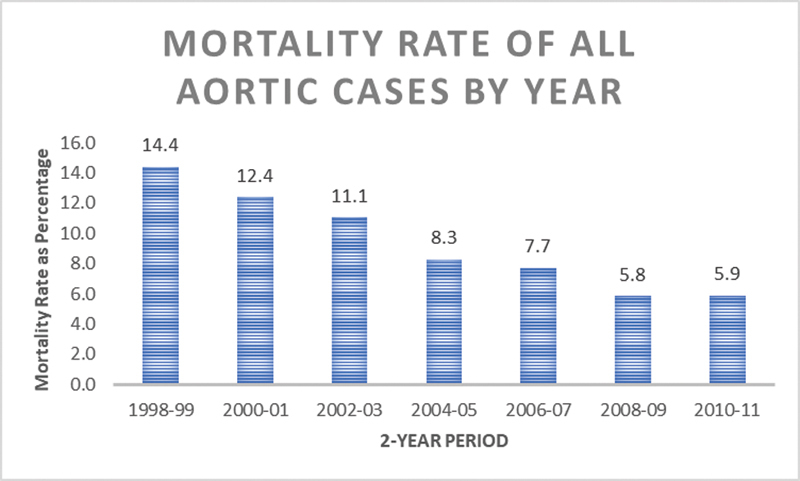
Decreasing mortality rate of all aortic cases (redo and nonredo) from 1998 to 2011.

**Fig. 4:**
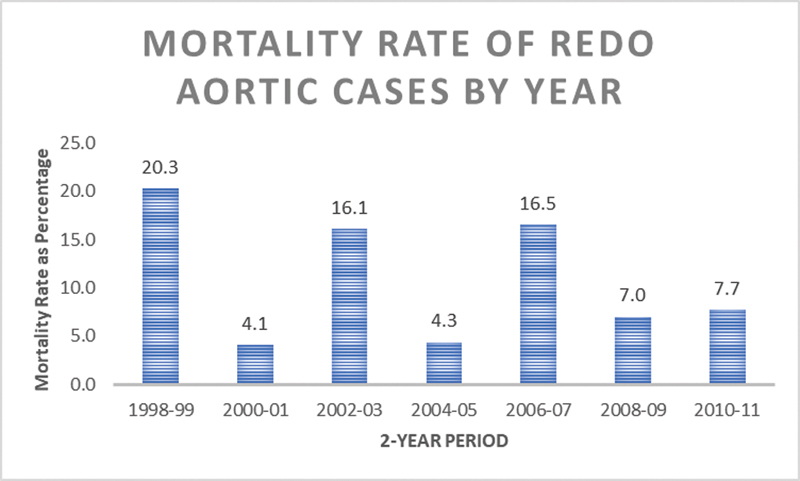
Mortality rate in redo patients from 1998 to 2011.

The number of patients receiving proximal thoracic aortic surgery for aortic dissection or aneurysm was determined for each year of the study period. We found that the overall number of proximal aortic surgeries sampled by the NIS has increased significantly over the study period, from 8,425 in 1998 to 21,837 in 2011, *p* < 0.05 ( Fig. 2 ). Further, the number of reoperative proximal aortic procedures has also increased significantly over the study period from 74 in 1998 to 1999 to 442 in 2011, *p* < 0.05 ( Fig. 1 ). At the same time, the in-hospital mortality rate of overall proximal thoracic aortic procedures decreased significantly during the study time period from 14.4% of all cases in 1998 to 1999 to 5.9% in 2010 to 2011, although the same trend was not seen in the reoperative group. The in-hospital mortality of redo proximal thoracic aortic procedures did not significantly decline over the study period. Trends in case volume and in-hospital mortality rate for both proximal aortic cases generally and redo proximal aortic cases specifically are summarized in Figs. 1 – 4 .

## Discussion

In this analysis of reoperative proximal thoracic aortic surgical outcomes in the United States, higher surgical volume is significantly correlated with lower in-hospital mortality.

### In-Hospital Outcomes

HVC and MVC had higher rates of pulmonary and gastrointestinal (GI) complications, as well as significantly higher rates of stroke compared with LVC. This is likely due to more complex patients undergoing surgery at higher volume centers. As detailed in Table 2 , patients seen at such centers have significantly higher baseline rates of chronic pulmonary disease and cerebrovascular disease. Patients at these institutions also underwent more concomitant coronary and valve procedures, increasing the complexity and morbidity of surgical repair.

### Risk of Reoperation in Cardiovascular Surgery

Reoperation in cardiovascular surgery generally has been associated with increased incremental mortality risk. The literature suggests, however, that outcomes comparable to patients undergoing first-time operations are achievable at centers that have designed the appropriate infrastructure and accumulated the requisite experience. Sabik et al 14 report of their experience with reoperative coronary surgery found that while redo patients may have higher mortality when compared with patients undergoing primary operations, reoperation itself was not significantly associated with mortality, but rather the patient's underlying risk profile was more impactful. 16 A study by Breglio et al 15 supported this conclusion by determining prior coronary surgery did not provide a unique risk factor for mortality in patients undergoing redo sternotomy for valve operations. 16 These reports from major cardiac surgery centers suggest that with the appropriate infrastructure and experience, reoperation itself need not introduce increased risk of mortality.

### Risk of Reoperative Proximal Aortic Surgery

Early reports of mortality after reoperative proximal aortic surgery from Crawford et al 3 demonstrated a 17% early mortality rate. Since this study, several major aortic referral centers have reported improved mortality in this challenging cohort. Girardi et al 4 reported initial excellent outcomes in his institution's series of reoperative proximal aortic surgical patients with a mortality rate of 5.4%. A follow-up report by Girardi et al 5 demonstrated even better mortality gained with experience, reporting a subsequent mortality rate of 4.1%. Di Bartolomeo et al 6 reported a series of 224 patients undergoing proximal thoracic aortic surgery who all had prior aortic surgery with an in-hospital mortality of 12%. In a series of patients undergoing redo root replacement and concomitant procedures, mortality was reported at 14%. 7 These series together suggest that while reoperative proximal thoracic aortic surgical patients, even in the best of hands, face considerable mortality risk, overall mortality is acceptable and improved with experience. In our study, the overall in-hospital mortality was 12%, which is within the range of most published reports of redo proximal thoracic aortic surgery. HVC had a staggeringly low operative mortality of 4.8%.

While accumulated experience appears to be correlated with improved outcomes, it is worthwhile to speculate about other causative factors. For example, the patient's prior operation, the specific redo operation, and extent of the aortic procedure, each plays a role. The group of patients who underwent redo root operations, for example, probably represent a high-risk cohort. In the series by Di Bartolomeo et al 6 reporting 12% mortality, 41% of patients had operations on their root, and Chen's entire cohort, 1 with 14% mortality, consisted of root patients. Unfortunately, the NIS database does not track prior operations or detail the extent of repair during the reoperation.

### Volume

Our study demonstrates that reoperative proximal thoracic aortic surgical patients overall have acceptable in-hospital mortality in the United States, but HVCs achieve significantly reduced in-hospital mortality. In subgroup analysis, patients undergoing elective redo aortic surgery had five times the odds of in-hospital mortality at an LVC compared with HVC. This further suggests that institutional experience directly impacts patient survival in reoperative proximal thoracic aortic surgery. Of note, while in-hospital mortality for all proximal thoracic aortic procedures is significantly decreasing over time, mortality for reoperative procedures is not. The decline in nonreoperative patients mimics that of other cardiac operations, and is likely driven by improved surgical technique, cerebral protection, and postoperative care. 17 Currently, two-thirds of patients undergoing reoperative proximal thoracic aortic surgery are operated on at centers that perform less than 40 cases annually, which is associated with higher in-hospital mortality.

Currently, there are no volume guidelines to guide referral of prior sternotomy patients who require proximal thoracic aortic surgery. Meanwhile, several studies have noted that redo aortic surgery volume appears to be increasing. A study by Luciani 8 revealed that the number of redo aortic procedures will likely rise as the population ages, as those with root prostheses and interventions for aortic dissection live longer. The study by Di Bartolomeo et al 6 revealed significant growth in their redo population as well. This upward trend in single-institution series was observed in our multicenter study, with 74 redo proximal thoracic aortic operations sampled in 1998 to 1999 rising to over 400 sampled in 2010 to 2011.

Volume standards have been developed for septal myectomy for hypertrophic cardiomyopathy, and mitral repair success rates in degenerative mitral valve disease have been shown to be highly correlated with annual surgeon volume. 18 As volume in redo aortic surgery increases, it appears worthwhile to consider whether instituting volume standards could improve overall outcomes in this complex patient population. Given the lack of decline in in-hospital mortality over the 14-year study, we advocate referral of reoperative proximal thoracic aortic patients to high-experience centers for the lowest odds of mortality.

### Limitations

There are several limitations to our study. First, this is a retrospective cohort study using the NIS, which is a national administrative database and is subject to coding error by nonclinical personnel. Second, the data included in this study only extends to the year 2011. The Nationwide inpatient sample is currently available from years 1998 to 2014. The way in which sampling is performed changed in the year 2012, making trend analysis of the entire dataset from 1998 to 2014 inappropriate. Several newer studies solely examine data from years 2012 to 2014 when looking at new procedures like transcatheter aortic valve replacement. The period, 1998 to 2011, was chosen because this period provided more data and standard techniques of redo sternotomy, and the associated aortic operations have generally remained standard. Another limitation, as mentioned above, is the paucity of information on the index operation performed, which means we are unable to separate patients who were likely to be more difficult reoperations. Further, we do not have information on the extent of the redo proximal thoracic aortic operation (i.e., root vs. ascending versus arch procedures). We also do not have information regarding cardioplegia strategy, cerebral protection, or bypass and cross-clamp times that may influence early morbidity and mortality. These limitations make the population more heterogeneous. Additionally, some surgeons may use cardiopulmonary bypass and hypothermia while performing descending thoracic and thoracoabdominal aortic operations, and these patients may not be effectively excluded by the search criteria. This study also does not capture survival beyond in-hospital mortality, need for reoperation, and freedom from the development of aortic insufficiency. Surgeon-specific volume may have provided additional insight into the impact of volume. Coding for this variable, however, is largely unreliable in the NIS. Therefore, we did not perform this analysis. Finally, mortality through NIS is only available as an in-hospital event. Thus, we are unable to report 30-day mortality that is utilized frequently the surgical literature.

## Conclusion

In this national study of reoperative proximal thoracic aortic surgery, we found that patients operated on at HVCs have significantly lower odds of in-hospital mortality than patients operated on at LVCs, both in the entire cohort and in the elective setting. Referral, when possible, to high-experience centers may reduce mortality in this high-risk group.
